# Design and Methodology of the Longitudinal Aging Study in India—Diagnostic Assessment of Dementia

**DOI:** 10.1093/geroni/igaa057.2279

**Published:** 2020-12-16

**Authors:** Jinkook Lee, Pranali Khobragade, Joyita Banerjee, Sandy Chien

**Affiliations:** 1 University of Southern California, Los Angeles, California, United States; 2 University of southern california, Los Angeles, California, United States; 3 All India Institute of Medical Sciences, New Delhi, Delhi, India

## Abstract

Longitudinal Aging Study in India is a nationally representative survey of the health, economic, and social wellbeing of the Indian population aged 45 and older. LASI-DAD is an in-depth study of late-life cognition and dementia, drawing a sub-sample of over 4,000 LASI respondents aged 60 or older. Respondents underwent a battery of cognitive tests, while their informants were interviewed about their cognitive and health conditions. A common set of cognitive tests was selected to enable international comparisons, and additional cognitive tests suitable for illiterate and innumerate populations were also selected. Rich data on risk factors of dementia were collected through health examinations, venous blood assays, and genotyping. The response rate was 82.9%, varying across sex, education, and urbanicity. LASI-DAD provides an opportunity to study late-life cognition and dementia and their risk factors in the older population in India and to gain further insights through cross-country analysis.

